# Acute kidney injury with extreme hyperuricemia after antithymocyte globulin treatment in a kidney transplant recipient with underlying aplastic anemia: a case report

**DOI:** 10.1186/s12882-020-01903-9

**Published:** 2020-07-02

**Authors:** Yohan Park, Byung Ha Chung, Cheol Whee Park, Yong-Soo Kim, Chul Woo Yang

**Affiliations:** Division of Nephrology, Department of Internal Medicine, Seoul St. Mary’s Hospital, College of Medicine, The Catholic University of Korea, 222 Banpo-daero, Seocho-gu, Seoul, 06591 South Korea

**Keywords:** Tumor lysis syndrome, Antithymocyte globulin, Aplastic anemia, Myelodysplastic syndrome, Kidney transplantation

## Abstract

**Background:**

The occurrences of hyperuricemia and acute kidney injury after antithymocyte globulin treatment are unusual in kidney transplant recipients. Here, we report a unique case of acute kidney injury with extreme hyperuricemia after antithymocyte globulin treatment in a kidney transplant recipient with underlying aplastic anemia.

**Case presentation:**

A 40-year-old woman with aplastic anemia who received a kidney transplant 5 years 6 months before presented to our emergency department with complaints of oliguria, generalized edema, and general weakness 6 days after receiving antithymocyte globulin treatment for acute T-cell-mediated rejection. Urinalysis revealed 100 uric acid crystal particles. The blood chemistry test results showed rapid increases in serum creatinine (from 2.86 mg/dL to 5.58 mg/dL) and uric acid levels (from 10.2 mg/dL to 32.7 mg/dL), which suggested acute uric acid nephropathy. Tumor lysis syndrome was suspected to be the cause of the acute uric acid nephropathy; hence, the patient was reevaluated for aplastic anemia. Human leukocyte antigen-DR15 was positive, and flow cytometry revealed a low percentage of glycophosphatidyl inositol-deficient granulocytes (2.9%), which suggested paroxysmal nocturnal hemoglobinuria clones. These findings indicate that the previously diagnosed aplastic anemia had either originally been hypocellular myelodysplastic syndrome (MDS) or later transformed into hypocellular MDS, which is a type of bone marrow failure syndrome.

**Conclusions:**

Clinicians should consider unexpected tumor lysis syndrome to be the cause of complications after antithymocyte globulin treatment in kidney transplant recipients with underlying bone marrow failure syndrome.

## Background

Acute uric acid nephropathy is one of the causes of acute kidney injury (AKI). Acute uric acid nephropathy is most commonly caused by tumor lysis syndrome (TLS); however, it may also occur from rhabdomyolysis or other conditions [1, 2]. When significant cell lysis occurs, large quantities of nucleotides are quickly released and metabolized in the liver, causing an increase in the serum uric acid level to > 12 mg/dL. Increased renal excretion of uric acid causes supersaturation in the urine, while the formation of uric acid crystals leads to the obstruction of the tubule lumen [3, 4].

TLS is usually caused by treatment of hematologic malignancies with high turnover rates, such as acute myeloid leukemia (AML), acute lymphoid leukemia (ALL), or lymphoma [5], and is relatively rare in bone marrow failure syndromes, which shows the hypocellularity of the bone marrow [6, 7]. Moreover, TLS caused by antithymocyte globulin (ATG) administration has not been reported in kidney transplant (KT) recipients to date. Here, we report a unique case of acute uric acid nephropathy presumably caused by ATG treatment-related TLS in a KT recipient with acute rejection who was previously diagnosed with aplastic anemia (AA).

## Case presentation

A 40-year-old woman who underwent kidney transplantation 5 years 6 months before presented to our emergency department with complaints of oliguria, generalized edema, and general weakness 6 days after receiving ATG treatment for acute T-cell-mediated rejection.

She was diagnosed with severe AA 8 years before on the basis of a bone marrow biopsy that revealed < 5% cellularity without cytogenetic abnormalities and with an inadequate response to ATG. Thus, she was managed conservatively with intermittent red blood cell and platelet transfusions without cyclosporine owing to concomitant chronic kidney disease. End-stage renal disease developed 7 years before, for which the patient had been receiving peritoneal dialysis (PD) for 1 year. She was transferred to our hospital for simultaneous kidney and hematopoietic stem cell transplantation. At that time, we planned a sequential transplantation (kidney transplantation followed by hematopoietic stem cell transplantation) with her mother as the donor. The ABO blood type was compatible, and two mismatched human leukocyte antigen (HLA) types were found. Panel reactive antibodies were 75% positive for class II. The donor-specific antibody was DR04, and the median fluorescence intensity was 1865. Rituximab (375 mg/m^2^) and basiliximab (20 mg) were administered as induction therapies. Tacrolimus, mycophenolate mofetil (MMF), and glucocorticoids were used for maintenance immunosuppression. However, MMF was changed to mizoribine after the patient experienced severe diarrhea and dyspepsia 3 years 6 months after receiving the KT.

**Fig. 1:**
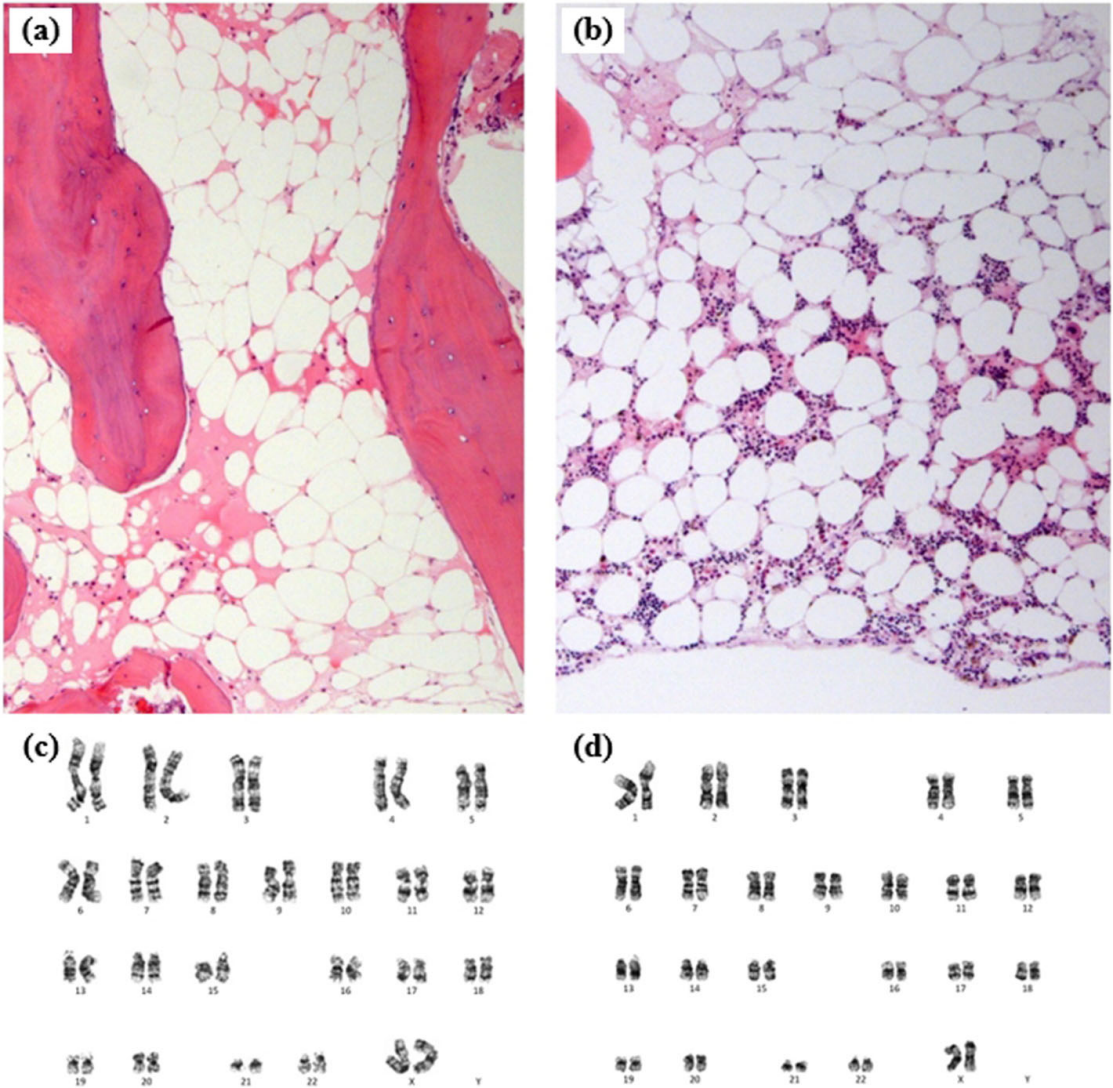
Bone marrow biopsy findings before and after the kidney transplantation. **a** Bone marrow biopsy findings before and (**b**) 16 months after kidney transplantation. The bone marrow cellularity of < 5% before the transplantation increased to 20% with normal hematopoietic differentiation. **c** Bone marrow biopsy karyotyping before and (d) 16 months after the kidney transplantation. Note the lack of cytogenetic abnormalities

**Fig. 2:**
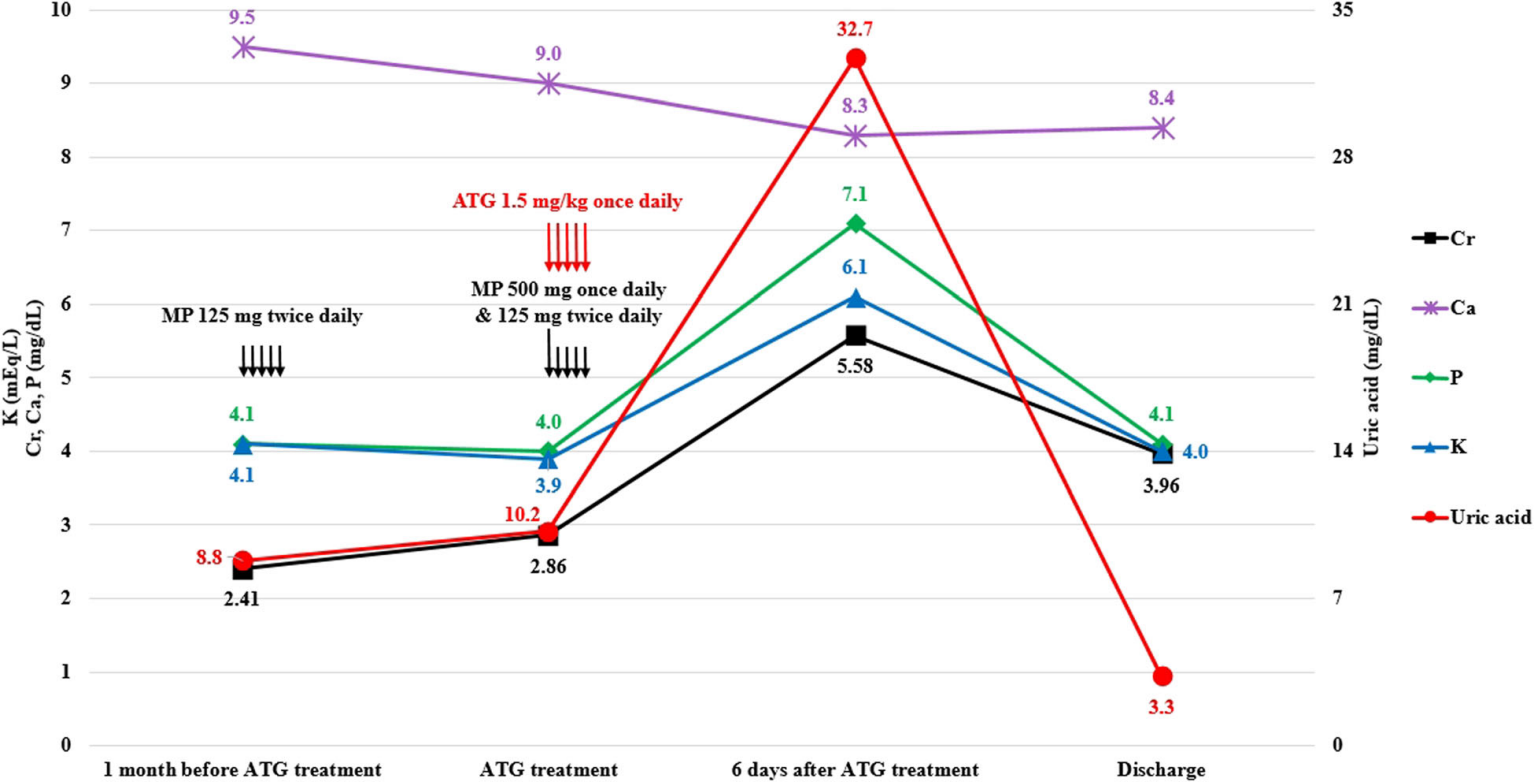
Laboratory findings before and after ATG administration and upon discharge. The uric acid level was remarkably increased after the ATG treatment for acute rejection, which suggests tumor lysis syndrome. Abbreviations: MP, methylprednisolone; ATG, antithymocyte globulin; Cr, creatinine; Ca, calcium; P, inorganic phosphorus; K, potassium

After kidney transplantation, the AA improved gradually without a hematopoietic stem cell transplantation. The complete blood cell count (CBC) improved with a hemoglobin (Hb) level of > 9 g/dL and platelet (PLT) count of > 50,000/μL, indicating that blood transfusion was unnecessary. The bone marrow biopsy findings improved 16 months after the kidney transplantation (Fig. 1). Graft function was also well-maintained; however, the serum creatinine (Cr) level increased gradually 5 years after kidney transplantation, reaching 2.41 mg/dL 5 years 5 months after transplantation. The graft biopsy revealed acute T-cell-mediated rejection type IA. We initially managed the patient with methylprednisolone pulse therapy (125 mg twice daily) for 5 days; however, her renal function deteriorated further after 1 month (Fig. 2). Thus, we administered ATG 1.5 mg/(kg·day) for 5 days. At that time, we pre-medicated the patient with methylprednisolone for 5 days, with 500 mg once and subsequently with 125 mg twice daily for the following 4 days.

On the day of admission, the patient complained of general weakness and generalized edema and showed a pretibial pitting edema. Blood testing revealed high serum blood urea nitrogen and Cr levels with hyperuricemia, hyperphosphatemia, and hyperkalemia, consistent with TLS. Urinalysis revealed > 100 uric acid crystal particles, and the uric acid-to-Cr ratio was 1.06, which was consistent with acute uric acid nephropathy [8] (Table 1).

**Table 1:** Laboratory findings at the emergency department at 6 days after antithymocyte globulin administration

**The complete blood cell count and serum chemistry test findings**	
WBC (× 10^3^/μL)	13.4
Hb (g/dL)	7.6
PLT (×10^3^/μL)	38
BUN (mg/dL)	73.1
Cr (mg/dL)	5.58
Uric acid (mg/dL)	32.7
Calcium (mg/dL)	8.3
Phosphorus (mg/dL)	7.1
Potassium (mEq/L)	6.1
Albumin (g/dL)	2.6
LDH (U/L)	556
CPK (U/L)	8
**The dipstick urinalysis and urine chemistry test findings**	
Hematuria	1+
Proteinuria	1+
Uric acid crystal (/HPF)	> 100
Uric acid (mg/dL)	46.4
Cr (mg/dL)	43.6
Uric acid / Cr ratio	1.06

Abbreviations: *WBC*, white blood cell; *Hb* Hemoglobin; *PLT* Platelet; *BUN* Blood urea nitrogen; *Cr* Creatinine; *LDH* Lactate dehydrogenase; *CPK* Creatine phosphokinase; *HPF* High power field

Two hemodialysis sessions were completed because of oliguria (urine output, < 200 mL/day). After hemodialysis, the patient’s serum uric acid and Cr levels remained at 3.3 and 3.96 mg/dL, respectively, with a daily urine output of > 1500 mL, and she was discharged (Fig. 2). TLS was suspected to be the cause of the acute uric acid nephropathy; therefore, post-transplantation lymphoproliferative disorder was considered a possibility. Epstein-Barr virus was not detected, and the imaging studies showed no findings indicative of lymphoma. Thus, the patient was reevaluated for the underlying hematologic disease. Flow cytometry of the paroxysmal nocturnal hemoglobinuria (PNH) clones revealed a 2.9% glycophosphatidyl inositol (GPI)-deficient granulocyte and a 2.9% CD24-deficient granulocyte expansion, which suggest that either the AA transformed into myelodysplastic syndrome (MDS) or the original underlying disease was MDS. Unfortunately, the patient progressed to graft failure 2 months after discharge and resumed PD.

## Discussion and conclusions

The present case demonstrates the occurrence of an extremely high increase in serum uric acid level (> 30 mg/dL) and AKI after ATG treatment in a KT recipient with underlying AA. We diagnosed the case as TLS based on the criteria for hyperuricemia, hyperphosphatemia, hyperkalemia, and AKI [9]. TLS usually presents within 7 days of cytotoxic chemotherapy [5] and most often occurs in hematologic malignancies with high turnover rates, such as AML and ALL, but is rarely reported in bone marrow failure syndromes such as AA and MDS [7, 9]. This is the first report of acute uric acid nephropathy presumably caused by ATG treatment-related TLS in a KT recipient with a previous diagnosis of AA.

TLS development after ATG treatment is unusual. This case was characterized by a long, 8-year history of AA with gradual improvement after kidney transplantation. Thus, the underlying AA was considered the cause of the TLS. A case review revealed two interesting findings. First, the patient was HLA-DR15 positive. In previous studies, AA patients with HLA-DR15 positivity showed 8.53 times higher hematologic improvement with immunosuppressants and higher coexisting PNH and MDS rates than the negative group [10, 11]. Second, the patient had PNH clones (2.9% GPI- and 2.9% CD24-deficient granulocytes), which suggested subclinical PNH. These features were consistent with those of a previous report that subclinical PNH in MDS patients showed a high HLA-DR15 positivity rate (90.5%) and bone marrow hypocellularity (64.3%). In addition, these patients often have normal karyotypic morphologies (95.2%) and respond well to immunosuppressants (77.8%) [12], as observed in the bone marrow biopsy findings from our case (Fig. 1). All the findings showed the possibility that the patient in this report either originally had hypocellular MDS or a previously diagnosed AA that had transformed into another type of bone marrow failure syndrome owing to clonal evolution [13, 14].

One may argue that high-dose methylprednisolone rather than ATG is responsible for the TLS in this case. Indeed, the development of TLS after high-dose methylprednisolone administration in MDS was previously reported [7]. In the review of our case, the patient was administered high-dose methylprednisolone two times with a 1-month interval (acute rejection treatment and premedication for ATG). If the high-dose methylprednisolone administration was responsible for the TLS in our case, TLS should have occurred during the first administration of methylprednisolone for acute rejection treatment. Thus, we suggest ATG, rather than methylprednisolone, to be the cause of the TLS, and that the possible pathophysiology of the ATG therapy-related TLS in MDS is the presence of potentially chemosensitive hematologic malignant cells. This presumption may be supported by a previous report of rapid tumor cell lysis occurring after ATG therapy for Sezary syndrome (cutaneous T-cell lymphoma) [15].

In this case, mizoribine was used as a maintenance immunosuppressant. Previous studies reported that mizoribine might cause hyperuricemia in patients with renal dysfunction [16, 17]. Mizoribine use may have contributed to the extreme hyperuricemia in this case; however, despite continuing the mizoribine therapy after TLS event, hyperuricemia was not observed anymore. Thus, mizoribine was unlikely to be the main cause of acute uric acid nephropathy in this patient.

This report has some limitations. First, we could not confirm the hematologic disease because the patient was reluctant to undergo bone marrow biopsy after developing TLS. Therefore, whether the patient’s underlying hematologic disease was MDS or AML was unclear. However, the patient’s CBC over the next 2 years after TLS event showed no evidence of blasts in peripheral blood that suggested AML. Second, whether the initial diagnosis of AA should have been hypocellular MDS is uncertain. However, our findings suggest that AA is a type of bone marrow failure syndrome that may overlap with other diseases or transform into another disease via clonal evolution during the disease course.

In conclusion, this was a unique case of ATG treatment-related TLS in a KT recipient previously diagnosed with AA. Thus, transplant clinicians should be cautious of the fact that the clinical course of AA may be more diverse than once thought and it can cause TLS in KT recipients receiving ATG treatment.

## Data Availability

Data sharing is not applicable to this article as no datasets were generated or analyzed during the current study.

## Abbreviations

AA: Aplastic anemia
AKI: Acute kidney injury
ALL: Acute lymphoid leukemia
AML: Acute myeloid leukemia
ATG: Antithymocyte globulin
CBC: Complete blood cell count
Cr: Creatinine
GPI: Glycophosphatidyl inositol
Hb: Hemoglobin
HLA: Human leukocyte antigen
KT: Kidney transplant
MDS: Myelodysplastic syndrome
MMF: mycophenolate mofetil
PD: Peritoneal dialysis
PLT: Platelet
PNH: Paroxysmal nocturnal hemoglobinuria
TLS: Tumor lysis syndrome
